# The retinal venous pressure at different levels of airway pressure measured with a new method

**DOI:** 10.1007/s00417-024-06483-0

**Published:** 2024-04-09

**Authors:** Johanna L. Baumgärtner, Richard Stodtmeister, René Mauer, Lutz E. Pillunat, Karin R. Pillunat

**Affiliations:** 1 Department of Ophthalmology, University Hospital Carl Gustav Carus Dresden, TU Dresden, Fetscherstr. 74, 01307 Dresden, Germany; 2 Institute for Medical Informatics and Biometry (IMB), Faculty of Medicine Carl Gustav Carus, TU Dresden, Fetscherstr. 74, 01307 Dresden, Germany

**Keywords:** Retinal venous pressure, Valsalva maneuver, Airway pressure, IOPstim, Glaucoma

## Abstract

**Purpose:**

This study is to investigate the increase in retinal venous pressure (RVP) induced by a stepwise increase in airway pressure (AirP) using the new IOPstim method, which is designed to artificially increase the intraocular pressure (IOP) and thus to stimulate vascular pulsation.

**Methods:**

Twenty-eight healthy subjects were examined in the left eye. The RVP was measured at baseline and at four different levels of AirP (10, 20, 30, and 40 mmHg) using the new IOPstim method: a half balloon of 8 mm diameter is inflated laterally to the cornea under observation of the central retinal vein. As soon as the vein pulsates at a certain AirP level, the IOP is measured with a commercially available tonometer, which then corresponds to the RVP.

**Results:**

Spontaneous venous pulsation was observed in all study participants. The mean RVP values at baseline and at the AirP levels of 10, 20, 30, and 40 mmHg were 17.6 ± 2.8 mmHg; 20.1 ± 3.0 mmHg; 22.1 ± 3.5 mmHg; 24.3 ± 3.7 mmHg, and 26.6 ± 4.2 mmHg, respectively. The mean RVP values of each AirP level were statistically significantly different from each other in pairwise comparison. In a linear mixed model, the effect of AirP on RVP was highly significant (*p* < 0.001). In the model, a 10-mmHg increase in AirP resulted in a linear increase in RVP of 2.2 mmHg.

**Conclusion:**

An increase in AirP was accompanied by a linear increase in RVP. The influence of AirP on RVP, and thus on retinal perfusion pressure during the Valsalva maneuver, is less than was assumed based on previous studies in which contact lens dynamometry was used.


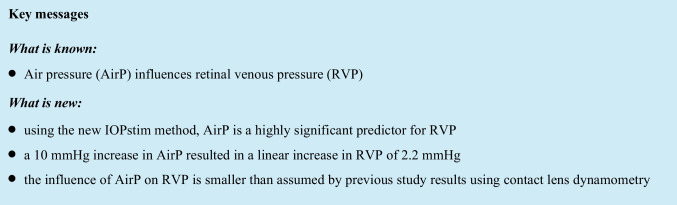


## Introduction

The measurement of retinal venous pressure (RVP) is required for correctly calculating ocular perfusion pressure (OPP = 2/3 mean arterial blood pressure minus RVP) [1]. A reduced perfusion pressure leads to impaired blood flow if the limits of autoregulation are exceeded [2, 3] or if autoregulation is disturbed [4]. This in turn results in ischemic and reperfusion damage [4]. Consequently, RVP is positively associated with visual field defects in glaucoma [5]. An elevation of RVP has been reported in various diseases, such as primary open-angle glaucoma [1] and diabetic retinopathy [6], but also in the Valsalva maneuver [7]. Heimann et al. showed an increase in RVP with a stepwise increase in airway pressure with the steepest increase at low airway pressures. They suggested that even small elevations in airway pressure, which occur frequently in everyday life, may negatively affect retinal perfusion pressure, and thus increase the risk of optic nerve damage [7]. In the mentioned study, RVP was measured with the contact lens dynamometer (CLD) according to Löw [8]. In this method, a commercially available Goldmann contact lens is placed on the cornea and the prevailing intraocular pressure (IOP) is calculated from the force required to trigger a pulsation phenomenon.

Recently, a new method of RVP measurement called IOPstim has been presented [9], in which a half-balloon of 8 mm diameter is placed on the ocular surface at the side of the cornea and inflated to artificially increase the IOP and thus to stimulate vascular pulsation. When the pulsation phenomenon of the central retinal vein or one of its branches occurs, the inflation is stopped and the IOP is measured with a commercially available tonometer. Due to the different measuring arrangements with varying vectors of force application, a comparison showed that RVP values measured with the IOPstim at a fixed level of AirP with 20 mmHg rendered 0.64 times the values of those with the CLD [10]. It can be assumed that the CLD method leads to an overestimation of RVP under the conditions of increased airway pressure. Therefore, Heimann et al. possibly overestimated the effect of airway pressure elevation on RVP. We hypothesized that the pattern of RVP increase would change under AirP increase for the same reason and tested this hypothesis in a diagnostic study [11]. This is the first published study to investigate the dependence of RVP on airway pressure using the new IOPstim method.

## Material and methods

A prospective cross-sectional study was conducted. This study was performed in line with the principles of the Declaration of Helsinki. Approval was granted by the Ethics Committee of the Technical University of Dresden (BO-EK-393082020).

Twenty-eight healthy subjects were examined on the left eye. Informed and written consent was obtained. The following inclusion criteria were defined: age from 18 to 40 years, written informed consent for voluntary study participation, pupil diameter dilatable to at least 6 mm.

Exclusion criteria were as follows: acute ocular inflammatory condition, acute corneal or retinal disease, myopia/spherical equivalent >|4.9|, condition after retinal detachment, corneal scars, postoperative ocular conditions, poor fundus imaging by optical media, diabetic retinopathy, ocular hypertension, glaucoma, optic nerve disease, upper arm circumference < 22 cm or > 42 cm, pregnancy or breastfeeding, known hypersensitivity or allergy to proxymetacaine hydrochloride or tropicamide, hypersensitivity to eye drops, no spontaneous pulsation of the central retinal vein or one of its branches on or in close vicinity of the optic disc (in the following called venous pulsation) present.

All examinations of a subject were performed by the same person during one appointment within 90 to a maximum of 120 min at the Department of Ophthalmology, University Hospital Carl Gustav Carus, TU Dresden.

The examination procedure was as follows:

First, best-corrected visual acuity and objective refraction (Oculus/Nidek AR-1 s, Oculus, Wetzlar, Germany) were determined. Then, baseline IOP was measured with a rebound tonometer (iCare PRO TA03, Icare Finland Oy, Vantaa, Finland). Next, the left pupil was dilated with tropicamide eye solution (5.0 mg/ml; Mydriaticum Stulln, Pharma Stulln GmbH, Stulln, Germany). In order to detect spontaneous venous pulsation (SVP), direct ophthalmoscopy (Beta 200 S ophthalmoscope, Heine Optotechnik, Gilching, Germany) was used. SVP was present in all study participants (100%). Subsequently, heart rate (HR) in beats per minute and blood pressure (BP) were determined by the oscillometric method (M500 automatic upper arm sphygmomanometer, Omron Healthcare Co., Ltd., Kyoto, Japan), and IOP in mydriasis. In the presence of SVP, this IOP corresponded to baseline RVP. After application of a local anesthetic (proparacaine POS 0.5% eye solution, URSAPHARM Arzneimittel GmbH, Saarbruecken, Germany), RVP was measured three times in quick succession at each level of AirP. The levels were set to 10, 20, 30, and 40 mmHg, randomized in the order of performance. There was a pause of 5 min between each level of AirP. Subjects increased AirP by forced expiration into an aneroid manometer (Aneroid Sphygmomanometer, Fazzini s.r.l., Vimodrone, Italy). RVP measurement was started no earlier than 3 s after the onset of AirP increase. The new IOPstim method was used for this purpose [9]. The IOPstim (IOPstim, Imedos Systems GmbH, Jena, Germany) resembles a spectacle frame with a half-balloon, which is applied to the eye laterally to the cornea. At each level of AirP, the pressure in this balloon was increased via a pneumatic system until venous pulsation was observed by funduscopy. At the onset of pulsation, the pressure increase was stopped and the artificially increased IOP was measured with the rebound tonometer. This IOP corresponded to the RVP. Subsequently, the pressure in the balloon was released. BP, HR, and IOP were also measured before each level of AirP and at the end of the study.

### Statistical analysis

Data were collected using Excel version 16.59 (Microsoft Corporation, Redmond, USA). The median of the three single measurements of RVP of each AirP level was used for further statistical analysis. Statistical calculations were performed using the program IBM SPSS Statistics for Macintosh (IBM Corporation, Armonk, USA) version 28.0.

Descriptive analysis included calculation of arithmetic mean (*M*) ± standard deviation (SD), minimum (min) and maximum (max), first quartile (Q1), median (Mdn), third quartile (Q3), interquartile range (IQR), absolute and relative frequencies, and 95% confidence intervals (95% CI).

The significance level of 5% was used for statistical hypothesis testing. The Shapiro-Wilk test and *Q*-*Q* plots were used to test for normal distribution. The coefficient of variation and the intraclass correlation coefficient (ICC) were determined as indicators of the intra-rater reliability of the three single measurements of RVP for each level of AirP. A linear-mixed effects model with random intercept and slope was applied with RVP as the dependent variable and AirP as the independent variable. Using the model, the pairwise comparisons of mean RVP values between the various AirP levels were conducted by *t*-tests with Bonferroni correction. Additional predictors were added to the model in stages, and their fixed effects were tested for significance using the *f*- or *t*-test. To examine whether the inclusion of a predictor resulted in a better fit of the model, the likelihood ratio test was performed, and the Akaike’s information criterions (AIC) were compared, whereby smaller values characterize better adaption [12, 13].

## Results

Twenty-eight subjects (20 female, eight male) aged 18 to 37 years (25.2 ± 4.5 years; *M* ± SD) were examined. The mean body mass index (BMI) was 22.2 ± 2.2 kg/m^2^. SVP was observed in all 28 study participants. Thus, the IOP in mydriasis was set equal to the baseline RVP. HR, diastolic, and systolic BP remained largely constant over the course of the study (Table 1).

**Table 1:** Parameters (mean ± standard deviation) before each level of airway pressure (AirP)

	Before AirP 10	Before AirP 20	Before AirP 30	Before AirP 40	Final
IOP [mmHg]	15.9 ± 3.5	16.2 ± 3.5	16.5 ± 3.2	15.9 ± 3.6	15.9 ± 4.2
HR [bpm]	73 ± 12	72 ± 10	72 ± 11	73 ± 12	72 ± 10
BPsys [mmHg]	115 ± 9	114 ± 9	115 ± 10	115 ± 9	113 ± 7
BPdia [mmHg]	77 ± 8	76 ± 10	77 ± 8	78 ± 9	76 ± 7

*IOP* intraocular pressure, *HR* heart rate, beats per minute, *BPsys* systolic blood pressure, *BPdia* diastolic blood pressure

The mean coefficient of variation of the three single measurements of RVP of each subject and the ICC for each AirP level are shown in Table 2. Measurement repeatability was very good [14].

**Table 2:** Coefficients of variations (mean ± standard deviation) and intraclass correlation coefficients (ICC) with 95% confidence interval (95% CI) for the three single measurements of retinal venous pressure (RVP) for each level of airway pressure (AirP)

RVP single measurements	Coefficients of variation	ICC [95% CI]
AirP 10	5 ± 2%	0.88 [0.79, 0.94]
AirP 20	5 ± 2%	0.90 [0.83, 0.95]
AirP 30	5 ± 2%	0.90 [0.83, 0.95]
AirP 40	4 ± 2%	0.90 [0.82, 0.95]

The RVP values at the different AirP levels were normally distributed. The mean RVP values at baseline and at the AirP levels of 10, 20, 30, and 40 mmHg were 17.6 ± 2.8 mmHg; 20.1 ± 3.0 mmHg; 22.1 ± 3.5 mmHg; 24.3 ± 3.7 mmHg; and 26.6 ± 4.2 mmHg, respectively (Table 3).

**Table 3:** Retinal venous pressure (RVP) at different levels of airway pressure (baseline, 10, 20, 30, 40 mmHg)

RVP [mmHg]	*M*	95% CI	SD	Min	Max	Q1	Mdn	Q3	IQR
Baseline	17.6	[16.5, 18.7]	2.8	11.8	22.4	15.9	18.4	19.7	3.8
AirP 10	20.1	[19.0, 21.3]	3.0	13.5	26.0	18.2	19.8	22.4	4.2
AirP 20	22.1	[20.8, 23.5]	3.5	15.9	28.7	20.0	22.3	24.5	4.5
AirP 30	24.3	[22.9, 25.7]	3.7	17.2	30.3	21.9	24.2	27.3	5.4
AirP 40	26.6	[24.9, 28.2]	4.2	18.2	35.1	24.4	26.6	29.2	4.8

*CI* confidence interval, *IQR* interquartile range, *M* mean, *Max* maximum, *Mdn* median, *Min* minimum, *SD* standard deviation, *Q1* first quartile, *Q3* third quartile

**Fig. 1:**
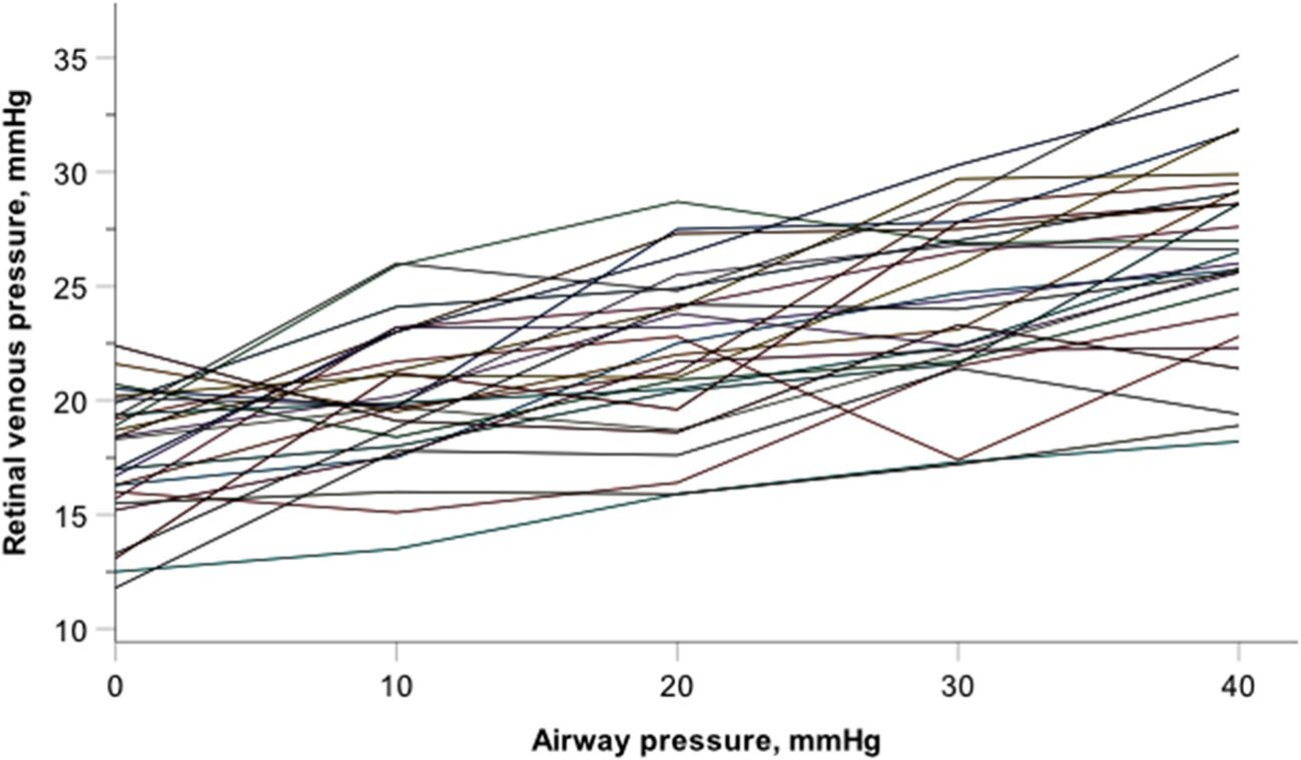
Retinal venous pressure as a function of airway pressure both in mmHg. The measured values of each subject (*n* = 28) are related with a line (graphics program used IBM SPSS Statistics for Macintosh, IBM Corporation, Armonk, USA, version 28.0)

The mean RVP values of each AirP level were statistically significantly different from each other in pairwise comparison (ΔAirP10-baseline, *p* < 0.001; ΔAirP20-AirP10, *p* = 0.011; ΔAirP30-AirP20, *p* = 0.005; ΔAirP40-AirP30, *p* = 0.002). The RVP values of all subjects as a function of AirP are shown in Fig. 1.

In the linear-mixed model, the fixed effect of AirP on RVP was highly significant (*p* < 0.001). In the model, a 10-mmHg increase in AirP resulted in a linear increase in RVP of 2.2 mmHg. When IOP was added as a covariate, the AIC decreased from 671 to 533. Thus, IOP before the respective AirP level had a significant fixed effect on the RVP of the respective AirP level (*p* = 0.042). The estimates of the best fitting model are shown in Table 4. The addition of other variables with fixed effects did not improve the fit of the model. Thus, sex, age, BMI, and spherical equivalent were not statistically significant predictors of RVP. No association with the systemic circulatory parameters HR, systolic, and diastolic BP could be shown either.

**Table 4:** Estimates of the best fitting mixed linear model

	Estimate	SE	df	*T*	95% CI	*p*
Intercept	15.48	1.33	100.20	11.68	[12.85, 18.11]	< 0.001
AirP	0.21	0.02	40.94	10.85	[0.17, 0.25]	< 0.001
IOP	0.15	0.07	101.67	2.06	[0.01, 0.30]	0.042

*AirP* airway pressure, *IOP* intraocular pressure, *SE* standard error, *df *degrees of freedom

## Discussion

The values of RVP obtained at the four levels of increased AirP are one-third smaller than those in the study by Heimann and co-workers [7], where RVP was measured with the CLD at the same levels of AirP. In another study [10] which compared the readings of both methods of RVP measurement at an AirP of 20 mmHg, the readings of the IOPstim method were also one-third lower. This ratio may therefore be attributed to the difference in measurement methods, and the reason should not be assumed to lie in the selection of subjects.

The smaller readings with the IOPstim method compared to CLD may be explained by the different directions of the force vectors during the examination [10]: In the CLD, the main force vector is directed towards the orbital apex, whereas this vector points towards the medial orbital wall in the IOPstim method. In the CLD, the eye is consequently moved towards the orbital apex. This increases the tissue pressure in this area, which then leads to a compression of the central vein. As a result, an increased outflow resistance is to be assumed. With the IOPstim method, the conditions are similar to the impression method [15], where the main vector is directed towards the medial orbital wall. Ulrich [16] has therefore recommended the suction cup method for measuring arterial pressures in the eye. The suction cup is placed on the surface of the eye laterally to the cornea. Then a negative pressure is induced. This increases the IOP without affecting the tissue pressure in the orbit. For this reason, the suction cup method is to be preferred for the measurement of arterial pressures in the eye. This method, however, is unsuitable for measuring the RVP because safe coupling to the sclera can only be assumed at a negative pressure of 50 mmHg [17]. This then causes an IOP increase of about 15 mmHg [17], which in many patients would lead to an IOP that is likely to be significantly higher than the threshold pressure of the RVP. Thus, of the available methods for measuring RVP, the IOPstim method is the one that can be presumed to have the lowest systematic error. It is remarkable that with the IOPstim device used here, the increase in RVP between the initial value and the first AirP level of 10 mmHg is much flatter than with the CLD method [7]. This difference may be explained by the different directions of the force vectors, as described above. Furthermore, the IOP increase caused by the coupling of the measurement device is significantly greater with the CLD at 10.0 mmHg than with the IOPstim method at 2.2 mmHg [10]. This leads to a large blind measurement range with CLD for low values of RVP.

Consequently, small increases in AirP, which can occur daily, have a smaller influence on RVP than could be assumed from previous measurements with the CLD.

In our study, there was a linear increase in RVP with an increase in AirP. This is consistent with the results of Korner et al. [18], who also presented a linear relationship between AirP and the invasively measured peripheral venous pressure in an upper arm vein [18]. It is safe to assume that the increased central venous pressure caused by the Valsalva maneuver [19] is transmitted via the jugular veins into the retinal veins [20]. Thus, according to our results, AirP is a significant predictor for RVP (*p* < 0.001). Consequently, an increase in airway pressure leads to a decrease in perfusion pressure via an increased RVP. The extent to which this leads to impaired perfusion depends on the limits and functionality of autoregulation. Among others, impaired autoregulation is discussed in patients with primary vascular dysregulation and glaucoma [21].

Thus, persistent or repeated increases in airway pressure, for example during chronic coughing or playing wind instruments, can potentially cause optic nerve damage in those affected. This hypothesis is supported by the results of Schuman’s group, according to which the cumulative playing time of a high-resistance wind instrument was significantly associated with an abnormal visual field [22]. Furthermore, the limits and mechanisms of retinal autoregulation in RVP alterations should be investigated in the future. So far, autoregulation has mainly been analyzed under modulation of systemic BP and IOP [21]. The extent to which the increase in RVP during a gradual increase in airway pressure affects the perfusion of the retina and optic nerve head may be the subject of future research. The results of the present study provide an estimate of the level of RVP at a given airway pressure. This will represent an essential basis for future studies on the influence of RVP on ocular blood flow. For this purpose, methods such as Fourier domain Doppler optical coherence tomography could possibly be used to measure ocular blood flow [23].

### Limitations of the study

It should not be ignored that some of the results were quite scattered. A major reason for this is that the venous system of the retina is influenced by the previously induced AirP level. Furthermore, it is probable that the participants become progressively more exhausted throughout the examination. To avoid a systematic error, the sequence of the AirP levels was randomized. Another reason for the variation might stem from the fact that the measurement of the RVP is a subjective method. However, the spread of the three single measurements of RVP was at most 2.2 ± 1.3 mmHg (*M* ± SD) at an AirP of 40 mmHg. The variation was smaller at the lower levels of AirP. Because of this relatively small variation, the results should not be rejected on the argument of subjectivity. To avoid investigator-dependent differences within the measurement series, all examinations were performed by the same person. Another limitation is that the number of subjects was not balanced with regard to gender. To test whether gender influences the RVP increase, sex was included as a predictor with fixed effect in the linear-mixed model. Adding gender as a factor resulted in AIC = 672. This value indicated a poorer fit of the model. This was also confirmed by the likelihood ratio test, *χ*2(1) = 0.23; *p* = 0.634. In conclusion, the fixed effect for gender was not statistically significant, *F*(1; 27.81) = 0.23; *p* = 0.635.

In conclusion, based on the results presented, the RVP can be estimated in the case of increased AirP. Previous results obtained with the CLD can now be compared quantitatively by conversion according to Table 5. The results shown here can be used as a basis for future studies, for example, to investigate retinal autoregulation in the context of increased RVP.

**Table 5:** Comparison of own results by IOPstim with those of Heimann et al. [1] by CLD

AirP [mmHg]	Baseline	10	20	30	40
Median RVP CLD [mmHg]	19.7	29.6	34.2	38.0	40.3
Median RVP IOPstim [mmHg]	18.4	19.8	22.3	24.2	26.6
IOPstim/CLD	0.93	0.67	0.65	0.64	0.66

*AirP* airway pressure, *CLD* contact lens dynamometry, *RVP* retinal venous pressure
